# Thymic papillary adenocarcinoma coexisting with type A thymoma: A case report

**DOI:** 10.1016/j.ijscr.2019.03.039

**Published:** 2019-03-30

**Authors:** Soichi Oka, Masaaki Inoue, Yohei Honda, Yasuhiro Chikaishi, Junichi Yoshida, Daisei Yasuda, Masao Tanaka

**Affiliations:** a Thoracic Surgery, Shimonoseki City Hospital, Shimonoseki, Japan; b Pathology, Shimonoseki City Hospital, Shimonoseki, Japan

**Keywords:** CT, computed tomography, VATS, video-assisted thoracic surgery, Mediastinal tumor, Thymic carcinoma, Thymoma

## Abstract

**Introduction:**

Thymic papillary adenocarcinoma is rare. Further, thymic papillary adenocarcinoma coexisting with type A thymoma is extremely rare. Surgery remains the only effective treatment for this disease.

**Presentation of Case:**

An 84-year-old Japanese woman presented to our institute due to abnormal chest computed tomography (CT) findings showing a 45 × 40 × 40-mm tumor located in the anterior mediastinum. A malignant tumor was suspected based on the CT findings and high serum levels of carcinoembryonic antigen. Mediastinal tumor resection was performed via video-assisted thoracic surgery through the left thoracic approach. This patient was discharged from our institute without any problems at six days post-operation.

**Discussion:**

This report has three major implications. First, one of the tumors was papillary adenocarcinoma. Primary papillary adenocarcinoma of the thymus is exceedingly rare. Second, papillary adenocarcinoma and type A thymoma coexisted in this tumor. Third, epithelial thymic tumor should be resected completely, since complete resection has been reported to be associated with an improved prognosis.

**Conclusion:**

We encountered a rare case of thymic papillary adenocarcinoma coexisting with type A thymoma.

## Highlights

• Thymic papillary adenocarcinoma is extremely rare.
• Further, thymic papillary adenocarcinoma coexisting with type A thymoma is extremely rare.
• Surgery remains the only effective treatment for this disease.
• We performed minimally invasive VATS approach.

## 1 Introduction

Thymic epithelial tumors, which include thymoma and thymic carcinoma, are rare cancers, with an annual incidence of approximately 0.15 cases in the United States and 0.32 cases in the Netherlands per 100,000 person-years [1,2].

Matsuno et al. suggested that papillary adenocarcinoma originates from type A thymoma. They reported that, in three of four cases, the components of papillary adenocarcionoma and spindle cell thymoma (type A thymoma) were present in the same nodule, and it is thought that papillary adenocarcinoma might arise from spindle cell thymoma as an expression of tumor progression or malignant transformation [3].

However, we herein report a case of papillary adenocarcinoma of thymic origin that coexisted separately with type A thymoma as a microscopic separate nodule. In this case, primary thymic adenocarcinoma existed independently from thymoma. Furthermore, we successfully performed complete local resection of this tumor via video-assisted thoracic surgery (VATS). This work has been reported in line with the SCARE criteria [4].

## 2 Presentation of case

**Fig. 1:**
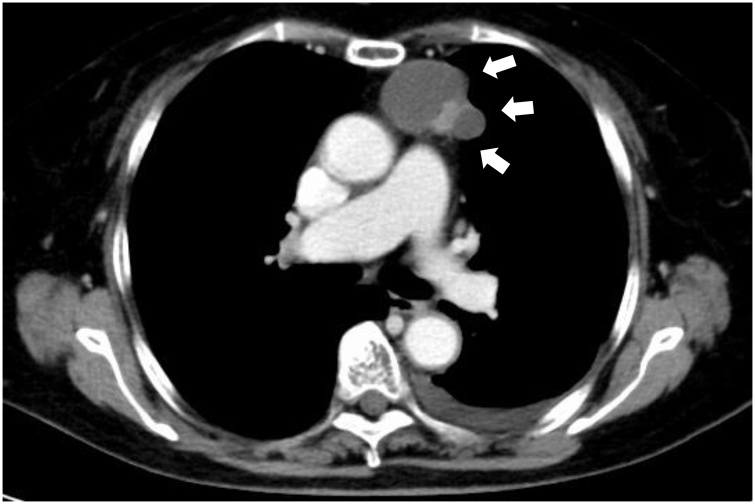
Computed tomography of the chest showing the localization of this tumor. This tumor was located in the anterior mediastinum and showed mixed cystic and solid lesions.

An 84-year-old Japanese women presented to our institute due to abnormal chest computed tomography (CT) findings showing a 45 × 40 × 40-mm tumor located in the anterior mediastinum. This tumor was composed of cystic and solid lesions (Fig. 1).

The patient had no remarkable medical history. Laboratory tests showed no abnormal findings but an elevated serum carcinoembryonic antigen (CEA) level, and abdominal CT also showed no abnormal findings. The mediastinum tumor was suspected to be malignant based on the CT findings and elevated CEA level; however, a definite pathological diagnosis for the tumor could not be made before the surgery. The tumor was resected local completely via VATS through the left thoracic approach. In the left thoracic cavity, a few small nodules were found. We resected these nodules. We confirmed local dissemination of thymic papillary adenocarcinoma with permanent specimen. This patient discharged healthy from our institute at six days post-operation.

**Fig. 2:**
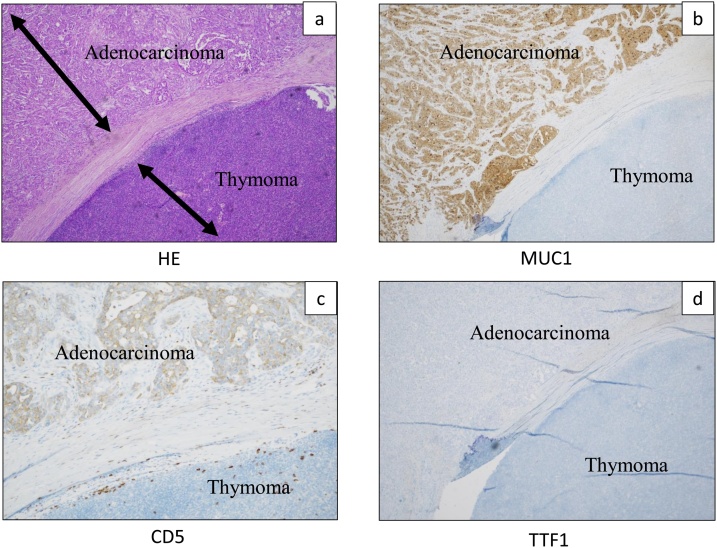
The pathological finding of this tumor. The tumor cells were mainly composed of papillary adenocarcinoma cells and type A thymoma (a). Immunohistochemical (IHC) staining was positive for MUC1 and CD5 (b and c) but negative for TTF-1 (d).

Pathologically, the tumor consisted of two separate components. These two microscopically separate tumors were diagnosed as a thymic papillary adenocarcinoma and a type A thymoma (Masaoka stage I) (Fig. 2a). The first tumor consisted of poorly differentiated papillary adenocarcinoma. There was no evidence of mucin production, nor were there any Hassal’s corpuscles around the tumor, but it was continuous with the thymic tissue. Immunohistochemical (IHC) staining was positive for MUC1, EMA, CD5, p63 and bcl-2 but negative for TTF-1, calretinin and thrombomodulin (Fig. 2b–d). The MIB-1 index was 70%. The second tumor was type A thymoma. This tumor consisted primarily of spindle-shaped epithelial cells and many lymphocytes without atypia (Fig. 2a).

## 3 Discussion

This report has three major implications. First, one of the tumors was papillary adenocarcinoma. Primary thymic carcinoma accounts for 14.1% of epithelial tumors of the thymus. Squamous cell carcinoma is the most common subtype of thymic cancer and accounts for 61.8%, and the frequency of thymic adenocarcinoma is only 1.6% [5]. Primary papillary adenocarcinoma of the thymus is exceedingly rare. Therefore, we must diagnose primary thymic adenocarcinoma carefully (In particular, exclusion diagnosis such as thyroid papillary adenocarcinoma). We carefully diagnosed using Immunohistochemical staining.

Second, papillary adenocarcinoma and type A thymoma coexisted in this tumor. There have been few reports of thymic carcinoma and thymoma coexisting [6]. Matsuno et al. suggested that papillary adenocarcinoma originates from type A thymoma. They reported that, in three of four cases, the components of papillary adenocarcinoma and spindle cell thymoma (type A thymoma) were present in the same nodule, suggesting that papillary adenocarcinoma might arise from spindle cell thymoma as an expression of tumor progression or malignant transformation [3]. However, our case was different, as primary thymic adenocarcinoma existed independently from thymoma. We could not detect these malignant transformation. Pathologically, we could not fine continuity in two tumors (thymic papillary adenocarcinoma and thymoma).

Third, epithelial thymic tumor should be resected completely, since complete resection has been reported to be associated with an improved prognosis. Indeed, we previously reported that the prognosis of completely resected thymic sarcomatoid carcinoma was good [7]. VATS is a useful approach, particularly for elderly patients. Li et al. reported that VATS was a safe and effective procedure for thymomas with a satisfactory prognosis [8]. We performed minimally invasive VATS in our patient, who was an 84-year-old woman. Our patient experienced no trouble with this operation and was discharged healthy from our institute six days after the operation. We plan to perform careful observation for this patient.

## 4 Conclusion

We encountered a rare case of thymic papillary adenocarcinoma coexisting with type A thymoma. Thymic adenocarcinoma has a poor prognosis, and its treatment has not been established. Further studies are warranted to collect more cases and elucidate the detailed characteristics of this tumor and identify targetable oncogenes.

## Conflicts of interest

We have no conflicts of interest.

## Sources of funding

We have no sources of funding for our research.

## Ethical approval

We got ethical approval from ethics committee of Shimonoseki city hospital, Japan.

## Consent

We had informed consent from this patient for writing this paper.

## Author contribution

Soichi Oka; study design, writing. Masaki Inoue; study design, other. Yohei Honda; other. Yasuhiro Chikaishi; other. Junichi Yoshida; other. Daisei Yasuda; other, Masao Tanaka; other.

## Registration of research studies

Yes, we can.

## Guarantor

Soichi Oka and Masaki Inoue

## Provenance and peer review

Not commissioned, externally peer-reviewed
